# Enhanced Crystallinity of Epitaxial Graphene Grown on Hexagonal SiC Surface with Molybdenum Plate Capping

**DOI:** 10.1038/srep09615

**Published:** 2015-04-24

**Authors:** Han Byul Jin, Youngeun Jeon, Sungchul Jung, Vijayakumar Modepalli, Hyun Suk Kang, Byung Cheol Lee, Jae-Hyeon Ko, Hyung-Joon Shin, Jung-Woo Yoo, Sung Youb Kim, Soon-Yong Kwon, Daejin Eom, Kibog Park

**Affiliations:** 1 School of Electrical and Computer Engineering, Ulsan National Institute of Science and Technology (UNIST), Ulsan 689-798, Republic of Korea; 2 Department of Physics, Ulsan National Institute of Science and Technology (UNIST), Ulsan 689-798, Republic of Korea; 3 School of Materials Science and Engineering, Ulsan National Institute of Science and Technology (UNIST), Ulsan 689-798, Republic of Korea; 4 Quantum Optics Laboratory, Korea Atomic Energy Research Institute, Daejeon 305-353, Republic of Korea; 5 Department of Physics, Hallym University, Chuncheon, Gangwondo 200-702, Republic of Korea; 6 School of Mechanical and Nuclear Engineering, Ulsan National Institute of Science and Technology (UNIST), Ulsan 689-798, Republic of Korea; 7 Korea Research Institute of Standards and Science, Daejeon 305-340, Republic of Korea

## Abstract

The crystallinity of epitaxial graphene (EG) grown on a Hexagonal-SiC substrate is found to be enhanced greatly by capping the substrate with a molybdenum plate (Mo-plate) during vacuum annealing. The crystallinity enhancement of EG layer grown with Mo-plate capping is confirmed by the significant change of measured Raman spectra, compared to the spectra for no capping. Mo-plate capping is considered to induce heat accumulation on SiC surface by thermal radiation mirroring and raise Si partial pressure near surface by confining the sublimated Si atoms between SiC substrate and Mo-plate, which would be the essential contributors of crystallinity enhancement.

Graphene is a 2D material composed of a monolayer of carbon atoms arranged in a honeycomb lattice structure^1^,^2^,^3^,^4^. Owing to its superior electron and hole mobilities, graphene has been considered to be a promising candidate material for ultra-fast electronic devices operating in THz frequency regime^5^. The first successful isolation of graphene was achieved by mechanically exfoliating highly oriented pyrolytic graphite (HOPG)^2^. Although high-quality single crystal graphene flakes can be obtained by mechanical exfoliation, the sizes of graphene flakes are too small (<100 μm) for practical applications^6^. Several alternatives including chemical vapor deposition (CVD)^7^,^8^, solid source deposition^9^,^10^, and surface graphitation of SiC^4^,^6^,^11^,^12^,^13^,^14^ have been explored for the synthesis of large-scale graphene. Of particular interest is the surface graphitation of a single crystalline SiC by thermal annealing in ultra high vacuum (UHV)^4^ or Ar environment^6^ at high temperature (>1300°C). In this process, only Si atoms are sublimated from the surface and the remaining C atoms rearrange to form a sample-size uniform so-called epitaxial graphene (EG) either on Si-face (0001) or C-face (000-1) surface^15^. The EG grown on C-face surface is normally thicker (typically 10–20 layers) than that on Si-face surface but its carrier mobility can reach as high as 18,700 cm^2^V^−1^s^−1^ 14. Hass *et al.* showed from first-principles calculations that such high carrier mobility of C-face EG is due to the unique rotational stacking faults residing in C-face EG^16^. These rotational stacking faults decouple the adjacent graphene layers electronically and make the multiple graphene layers maintain the electronic properties of an isolated single layer graphene. Very recently, Trabelsi *et al.* have reported that a few or even single layer of graphene could be grown epitaxially on C-face surface in the form of islands (hundreds of μm) or freestanding bubbles (several μm)^17^,^18^. Their results imply that it is possible to control the thickness of EG grown on C-face surface by carefully adjusting the Si flux supplied externally and the growth time during the conventional UHV annealing. Based on the large-scale availability and good electrical properties, the EG on SiC surface (either Si-face or C-face) clearly demonstrates the potential to be used as a platform for future electronic devices. However, it is necessary to work continuously on lowering the formation temperature of EG while maintaining its superior electrical properties in order to fabricate high-performance electronic devices at reduced processing costs. This is quite crucial for the actual commercialization of EG-based electronics in competition with the current Si technology. In this work, we have developed an experimental method to significantly improve the crystallinity of EG grown on a Hexagonal-SiC substrate simply by capping the substrate with a molybdenum plate (Mo-plate) during UHV annealing.

## Results

### Growth of EG films on n-type C-face 4H-SiC surface with Mo-plate capping and structural analyses

**Figure 1:**
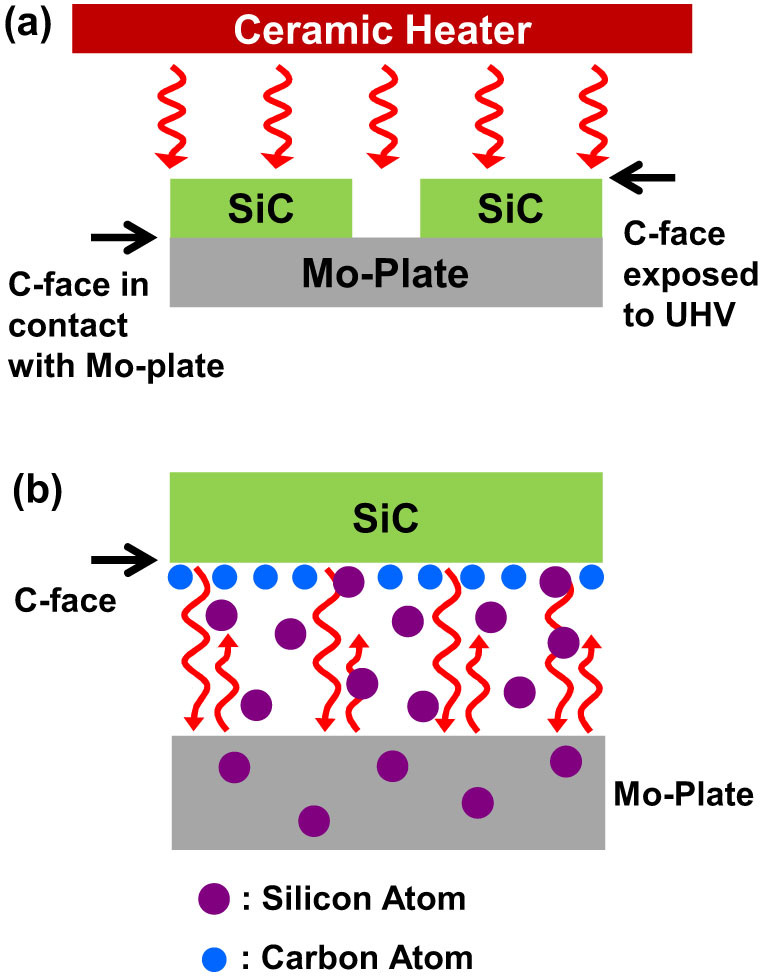
(a) Schematic view of the experimental configuration to perform annealing C-face Hexagonal-SiC surface in UHV *with* (left) and *without* (right) Mo-plate capping simultaneously. The red curvy lines represent the thermal radiation emitted from the ceramic heater and delivered to the assembly of the SiC substrates and the Mo-plate. (b) The zoom-in of the region where the C-face of Hexagonal-SiC is in contact with the Mo-plate. The red curvy lines represent the thermal radiation emitted from the Hexagonal-SiC substrate and reflected from the Mo-plate surface. From XPS measurements, the silicon atoms (Purple filled circles) sublimated from the 4H-SiC surface are found to be absorbed and diffuse into the Mo-plate.

The EG film was first grown on an n-type C-face 4H-SiC substrate 4-degree miscut to <11–20>. The SiC substrate was chemically cleaned with HF (49%) for 1 min followed by methanol rinse to remove native oxides. The Mo-plate was also cleaned with HCl:H_2_O (2:1) solution for 10 min followed by DI rinse and annealing at 500°C in UHV to remove the residues from machining processes. In order to compare the growth of EG *with* and *without* Mo-plate capping, the C-face surface of one 4H-SiC sample was in contact with the Mo-plate while that of the other 4H-SiC sample was exposed to UHV environment during annealing as shown in Figure 1. The samples prepared this way were annealed for 10–60 min at 850–950°C, which is substantially lower than the conventional vacuum annealing process. The temperature was measured by using both an IR pyrometer and a thermocouple for cross-checking. The chamber base pressure was 6.0 × 10^−9^ Torr and the working pressure became as high as ~4.6 × 10^−6^ Torr when the annealing time reached 60 min at 900°C.

**Figure 2:**
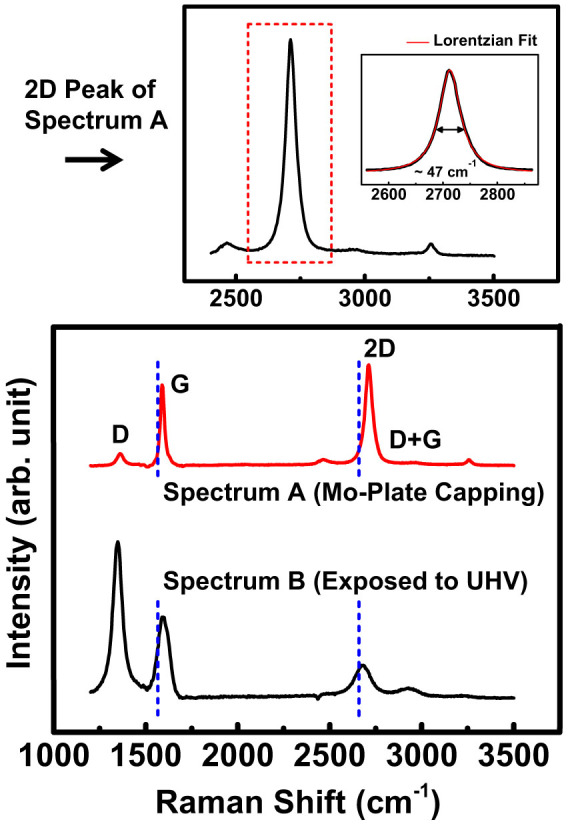
Raman spectra of EG films grown on n-type C-face 4H-SiC *with* (Spectrum A) and *without* (Spectrum B) Mo-plate capping. The blue-dotted lines represent the positions of G and 2D peaks for a mono-layer graphene obtained by mechanical exfoliation. For clear comparison, the measured Raman spectra are scaled so that the G peak heights of Spectrum A and Spectrum B are identical to each other. The upper graph is the zoom-in of the 2D peak of Spectrum A (Mo-plate capping) with a single Lorentzian fit where the fitted FWHM is ~47 cm^−1^.

In order to confirm the formation of EG and evaluate its structural quality, Raman spectrum measurements were performed by using a Raman microscope (Alpha 300R, WITec). The laser excitation energy was ~2.33 eV (532 nm), the laser power 3 mW, and the laser spot size ~ 1.2 μm. Figure 2 shows the Raman spectra of the EG films grown on an n-type C-face 4H-SiC substrate at 900°C *with* (Spectrum A) and *without* (Spectrum B) Mo-plate capping, which were acquired by subtracting the background signal coming from the 4H-SiC substrate. The characteristic Raman peaks (D, G, 2D) of graphene are seen clearly in the spectra for both cases, indicating the actual formation of EG. One apparent difference between the two spectra is that the relative intensities of D and 2D peaks with respect to G peak vary significantly. In case of the EG film grown *without* Mo-plate capping, the D peak intensity is ~1.9 times the G peak intensity while the 2D peak intensity is ~0.4 times the G peak intensity. A noticeable size of D+G peak that is directly linked to D peak is also observed. These indicate that the formed EG film is defective and the sizes of graphene grains are quite small (very likely nm scale)^19^, which is expectable for the EG film grown at such relatively low temperature in UHV. In contrast, the D peak intensity of the EG film grown *with* Mo-plate capping is quite small, ~0.1 times the G peak intensity, and the 2D peak intensity is ~1.25 times the G peak intensity. The D+G peak is very small to be almost negligible. These changes in Raman spectra indicate that the EG film grown *with* Mo-plate capping contains larger grains with less defects in it, compared with the one grown *without* Mo-plate capping. Thus, Mo-Plate capping makes the crystallinity of the EG film grown at relatively low temperatures almost as good as the film grown at normal high temperatures in UHV. Here, we note that the measured temperatures represent how hot the Mo-plate and SiC surface become by the thermal energy supplied from the heater when they are exposed to UHV (Figure 1). The temperature in the region between the Mo-plate and the SiC substrate is not directly measurable and can be different from the value measured with the pyrometer or the thermocouple.

Another point noteworthy in the measured Raman spectra goes with the positions and shapes of G and 2D peaks. An EG film is generally known to have its G peak blue-shifted due to the residual compressive strain arising from the interaction between substrate and EG film^20^,^21^,^22^. In the measured Raman spectra, the G peaks of the EG films grown *with* and *without* Mo-plate capping are located at ~1588 cm^−1^ and ~1596 cm^−1^ respectively, which are indeed blue-shifted with respect to the G peak(~1580 cm^−1^) of a mechanically exfoliated mono-layer graphene. As the EG film gets thicker, however, the compressive strain relaxes and the associated blue-shift of G peak becomes smaller. Hence, the smaller blue-shift of the EG film grown *with* Mo-plate capping signifies that the film is thicker than the one grown *without* Mo-plate capping. This interpretation is also supported by the position of 2D peak. The 2D peak of the EG film grown *without* Mo-plate capping is positioned at ~2680 cm^−1^, typical for thin EG films grown on C-face hexagonal SiC^20^. Whereas the 2D peak of the EG film grown *with* Mo-plate capping is positioned at ~2711 cm^−1^, more shifted toward the bulk graphite value^21^,^22^ of ~2725 cm^−1^, and this is a clear indication of the EG film grown *with* Mo-plate capping being thicker. The shape of 2D peak provides the information about the rotational ordering in the stacking of graphene layers. As shown in Figure 2, the 2D peak of the EG film grown *with* Mo-plate capping is quite symmetric, differently from bulk graphite, and fitted well to a single Lorentzian with a full width at half maximum (FWHM) of ~47 cm^−1^, larger than the typical value (~24 cm^−1^) for a monolayer graphene. This suggests that the stacking of graphene layers is not AB-Bernel one rather rotationally random with respect to one another, so called turbostratic^21^,^22^. The rotational disorder in the stacking of graphene layers is observed frequently in the EG films grown on C-face surface^23^.

**Figure 3:**
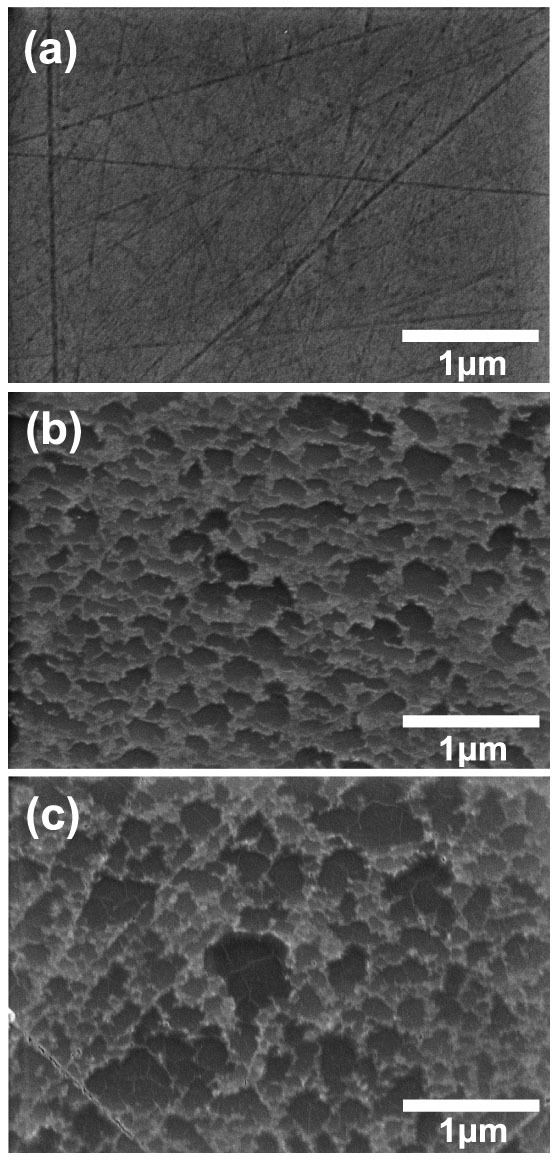
SEM images taken on the surface of n-type C-face 4H-SiC substrate annealed for 1 hr (a) at 900°C with the surface exposed to UHV (*without* Mo-plate capping), and at (b) 850°C and (c) 900°C *with* Mo-plate capping.

Scanning electron microscope (SEM) images were taken to investigate the surface morphology of the grown EG films by using a Hitach Cold FE-SEM. Figure 3(a) shows the surface of the 4H-SiC substrate exposed to UHV during annealing at 900°C, whose texture is almost identical to that of the bare 4H-SiC surface before annealing. This implies that the surface graphitation of the 4H-SiC substrate occurs only in a couple of atomic layers near the surface and localized in narrow regions so as not to cause any noticeable change of the surface morphology in large scale. On the other hand, the 4H-SiC surface annealed at 850°C *with* Mo-plate capping looks much rougher than the bare 4H-SiC surface (Figure 3(b)). It is believed that this surface roughness is due to the step bunching driven by the sublimation of Si atoms and the active surface migration of the remaining C atoms during EG formation. The step bunching on the surface is observed to be enhanced further at higher temperature (900°C), making the area bounded by bunched steps to become larger, as shown in Figure 3(c). This enhancement of step bunching at higher temperature is consistent with the common notion that the sublimation of Si atoms and the surface migration of C atoms will increase as temperature increases.

### Mechanism of crystallinity enhancement of EG films grown with Mo-plate capping

Both Raman spectra and SEM images suggest that the Mo-plate capping provides a unique kinetic environment to grow high crystallinity EG films at substantially reduced temperatures. It is quite interesting and important to address how the Mo-plate capping gives rise to such structural improvement of EG films. Although it has not been verified in quantitative manners, the following two mechanisms are considered to be the main contributors. Firstly, since the 4H-SiC substrate and the Mo-plate just make a physical contact with each other, it is naturally expected to have a microscopically narrow gap between them. When the 4H-SiC substrate is heated, thermal radiation will be emitted from the substrate in all directions. Here, the thermal radiation emitted from the 4H-SiC surface in contact with the Mo-plate could be strongly reflected from the Mo-plate surface because of the metallicity of Mo as illustrated in the zoom-in in Figure 1. This *radiation mirroring* from the Mo-plate will prevent thermal loss and induce heat accumulation on the 4H-SiC surface. Accordingly, the temperature of the 4H-SiC surface in contact with the Mo-plate could be higher than the opposite surface exposed to UHV with thermal loss by radiation. Therefore, the buried 4H-SiC surface in contact with the Mo-plate might reach temperature high enough to bear the active Si sublimation and C atom surface migration for full growth of EG although the thermal energy from the heater is not sufficient to do so for the 4H-SiC surface exposed to UHV. That is to say, while the 4H-SiC surface exposed to UHV reaches only the temperature range of 850–950°C (IR pyrometer and thermocouple readings as described previously), the temperature of the buried 4H-SiC surface in contact with the Mo-plate could be much higher. Secondly, as the Si sublimation progresses on the 4H-SiC surface, the Si vapor density in the narrow gap will increase. Several previous studies^6^,^24^,^25^,^26^,^27^ have reported that the confinement of Si vapor in the vicinity of SiC surface significantly reduces the growth rate of EG by triggering the condensation of sublimated Si atoms back onto SiC surface and the reduction of growth rate leads to the formation of uniform EG with well-controlled thickness. Along the same line as these reports, the increase of Si vapor density in the gap would slow down the net sublimation of Si atoms and establish an environment favorable to grow high-quality EG films.

**Figure 4:**
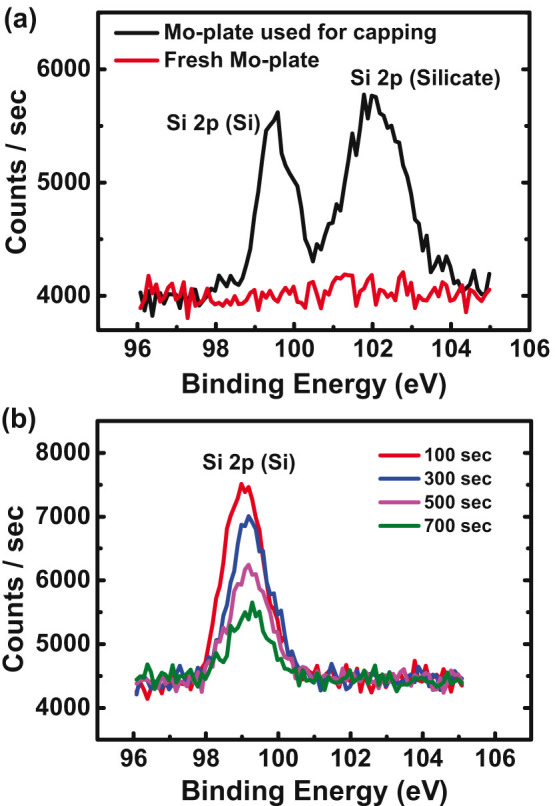
(a) XPS spectra measured on the Mo-plate used for capping the SiC surface and a fresh Mo-plate: Si 2p signals are seen only on the Mo-plate used for capping in the form of pure Si and silicate. (b) Si 2p XPS spectra taken on the Mo-plate used for capping while etching off the Mo-plate from the surface into the bulk: The intensity of Si 2p signal decreases as going further into the bulk, indicating that the Si atoms sublimated from the SiC surface are absorbed and diffuse into the Mo-plate.

However, as Çelebi *et al.* pointed out^24^, the sublimated Si atoms should be drained out of the gap in order to kick off the formation of EG. Otherwise, the sublimation and condensation rates will equilibrate to have no net sublimation of Si atoms. Unlike the previous studies^6^,^24^,^25^,^26^,^27^ mentioned above, the flat Mo-plate covers the entire 4H-SiC surface in our case and hence there is no well-defined pathway through which the sublimated Si atoms in the gap can escape to UHV. Surprisingly, we have found that the Mo-plate itself plays a role of a drain or sink, that is, the sublimated Si atoms are absorbed and diffuse into the Mo-plate (Zoom-in in Figure 1). This is verified from the X-ray Photoelectron Spectroscopy (XPS) measurements where the intensity of Si 2p peak is traced from the surface into the interior of the Mo-plate (Figure 4). Figure 4(a) shows the XPS spectra measured on the surface of the Mo-plate used for capping and a fresh Mo-plate. As shown in the figure, Si 2p signals are observed only on the surface of the Mo-plate used for capping, which are contributed from both pure Si and silicate^28^. The silicate signal is understandable since the Mo-plate was exposed to air before measurement, prompting some Si atoms to get oxidized. Figure 4(b) shows the XPS spectra measured while etching off the Mo-plate used for capping with Ar ions. As seen in the figure, Si atoms reside even inside the Mo-plate though their concentration decreases as going into the bulk. Since Si 2p signal is not observed in the fresh Mo-plate, the Si atoms detected in the capping Mo-plate must be the ones sublimated from the 4H-SiC surface. In other words, Mo-plate absorbs the Si atoms sublimated from the 4H-SiC surface to facilitate a nonzero net sublimation.

### Growth of EG films on semi-insulating Si-face 6H-SiC surface and characterization of structural and electrical properties

**Figure 5:**
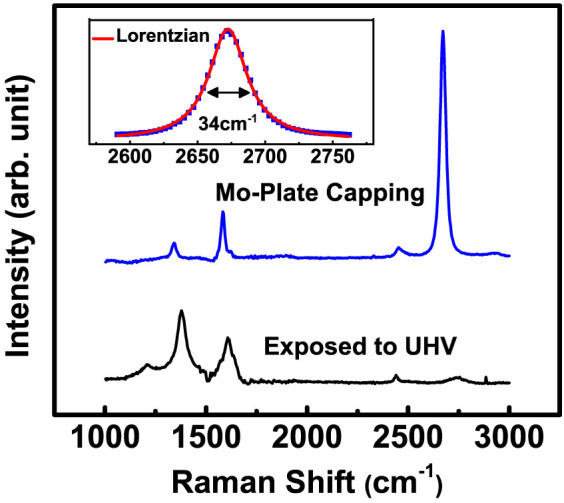
Raman spectrum of a EG film grown on a Si-face semi-insulating 6H-SiC substrate *with* and *without* Mo-plate capping: The inset is the zoom-in of 2D peak of the EG film grown *with* Mo-plate capping together with the single Lorentzian fit where the fitted FWHM is ~34 cm^−1^.

**Figure 6:**
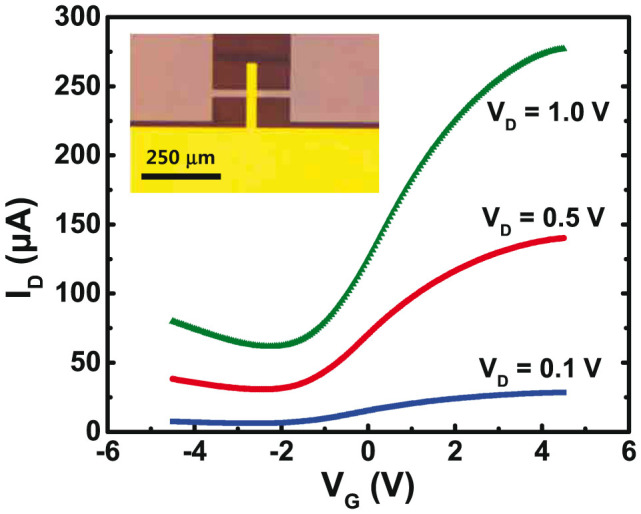
The drain current vs. gate voltage curves of a top-gated FET fabricated by using the EG film grown on a Si-face semi-insulating 6H-SiC surface for several different drain voltages. The inset is an optical microscope image of the fabricated FET. The field-effect mobility is estimated to be ~1800 cm^2^/Vs at the drain voltage of 0.1 V.

The C-face n-type 4H-SiC substrate was not suitable for characterizing the electrical properties of EG films grown on it because of the leakage current through the doped substrate and a significant amount of polishing scratches on surface (Figure 3). Unfortunately, the UHV system used for our experiments was not equipped with a high-temperature hydrogen etching apparatus capable of removing polishing scratches. Hence, a semi-insulating Si-face on-axis 6H-SiC substrate was used for electrical measurements on EG films, which was supplied from the vendor with only the Si-face surface removed of polishing scratches (The C-face surface contained a large amount of polishing scratches). The EG films on Si-face 6H-SiC surface were grown in the exactly same manner as the C-face 4H-SiC case (Figure 1) at temperature 950°C. Based on the Raman spectra shown in Figure 5, the structural quality of the EG film grown *with* Mo-plate capping is found to improve greatly in comparison with the one grown *without* Mo-plate capping. The ratio of 2D peak to G peak (~5:1) and the FWHM of 2D peak (~34 cm^−1^) indicate that the EG film grown *with* Mo-plate capping is likely to be a monolayer. The electrical properties of the EG film were measured by fabricating top-gated field effect transistor (FET) devices. Figure 6 shows the channel current of one of the fabricated FETs as a function of gate voltage for several different drain voltages. As shown in the figure, the charge neutrality point is negative, meaning that the carrier type of the as-grown EG film is n-type. From the measured current-voltage characteristics, the field-effect mobility^29^ is estimated to be ~1800 cm^2^/Vs at the drain voltage of 0.1 V. This estimated field-effect mobility is somewhat higher than the values reported previously in other researches for Si-face EG grown by the conventional high-temperature vacuum annealing^30^,^31^, ranging from 200 to 1200 cm^2^/Vs. The detailed information for FET fabrication^32^ and mobility extraction procedure is available in Supplementary Information. The measured FET characteristics imply that our EG films grown *with* Mo-plate capping possess good electrical properties consistent with the high structural quality.

## Conclusions

In summary, we report that the structural crystallinity of EG films grown on a Hexagonal-SiC substrate can be improved greatly by capping the substrate with a Mo-plate during UHV annealing. The effect of Mo-plate capping is considered to be two-fold. The first is that the Mo-plate induces heat accumulation on the SiC surface in contact with it by reflecting thermal radiation from the surface. The second is that the Mo-plate confines the Si atoms sublimated from the SiC surface in the narrow gap between the SiC substrate and the Mo-plate and absorbs them. These two phenomena can cooperatively facilitate an environment favorable for growing high-quality EG films where the net Si sublimation is nonzero but small enough to slow down the growth rate of EG. With no need to heat the entire SiC substrate to high temperature over 1300°C, the Mo-plate capping can be an easily-applicable experimental method to reduce energy consumption greatly in growing high quality EG films.

## Supplementary Material

Supplementary Information Supplementary Information
