# Supplementary material for: Enhanced Crystallinity of Epitaxial Graphene Grown on Hexagonal SiC Surface with Molybdenum Plate Capping
Source: Sci Rep. 2015 Apr 24;5:9615. doi: 10.1038/srep09615 (PMC5386107; doi:10.1038/srep09615)
Supplement: Supplementary Information [file srep09615-s1.doc]

**Supplementary Information**

**Enhanced Crystallinity of Epitaxial Graphene Grown on Hexagonal SiC Surface with Molybdenum Plate Capping**

Han Byul Jin^1^, Youngeun Jeon^1^, Sungchul Jung^2^, Vijayakumar Modepalli^3^, Hyun Suk Kang^4^, Byung Cheol Lee^4^, Jae-Hyeon Ko^5^, Hyung-Joon Shin^3^, Jung-Woo Yoo^3^, Sung Youb Kim^6^, Soon-Yong Kwon^3^, Daejin Eom^7^, and Kibog Park*^1,2^

^1^School of Electrical and Computer Engineering, Ulsan National Institute of Science and Technology (UNIST), Ulsan 689-798, Republic of Korea

^2^Department of Physics, Ulsan National Institute of Science and Technology (UNIST), Ulsan 689-798, Republic of Korea

^3^School of Materials Science and Engineering, Ulsan National Institute of Science and Technology (UNIST), Ulsan 689-798, Republic of Korea

^4^Quantum Optics Laboratory, Korea Atomic Energy Research Institute, Daejeon 305-353, Republic of Korea

^5^Department of Physics, Hallym University, Chuncheon, Gangwondo 200-702, Republic of Korea

^6^School of Mechanical and Nuclear Engineering, Ulsan National Institute of Science and Technology (UNIST), Ulsan 689-798, Republic of Korea

^7^Korea Research Institute of Standards and Science, Daejeon 305-340, Republic of Korea

**The fabrication processes and characterization of FET devices using epitaxial graphene (EG) films grown on a Si-face semi-insulating 6H-SiC surface**


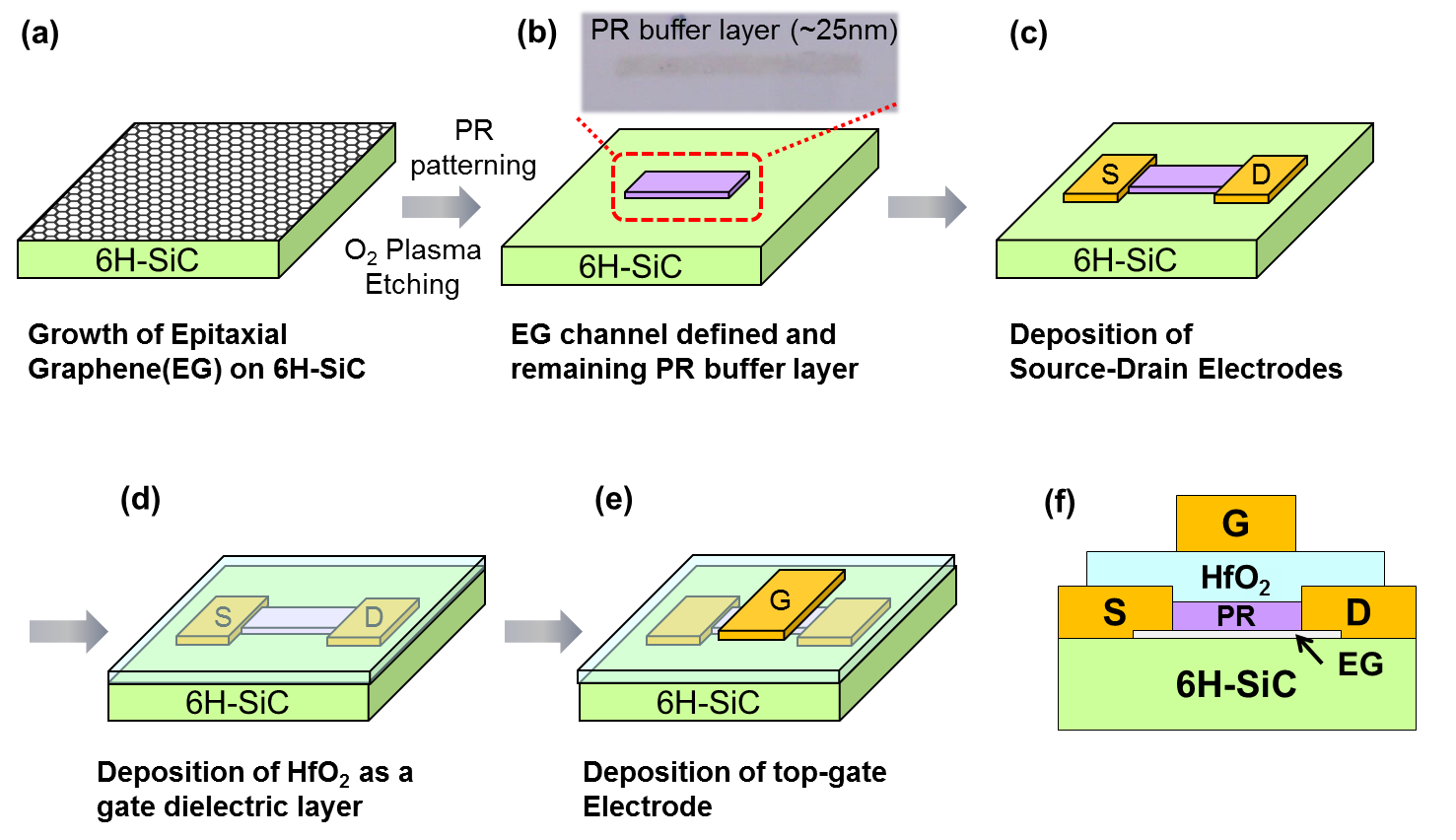


**Figure S1.** Schematic diagram of the fabrication processes to obtain a top-gated EG FET on Si-face semi-insulating 6H-SiC. (a) Wafer size EG is grown on 6H-SiC surface with Mo-plate capping (b) O_2_ plasma etching is employed to pattern the EG channel after photolithography step. The photoresist residue remaining after acetone cleaning is used as a buffer layer for depositing the gate dielectric layer. (c) The source-drain electrodes are deposited by e-beam evaporation. (d) The HfO_2_(~50nm) gate dielectric layer is deposited by using ALD. (e) Finally, the top gate electrode is deposited by e-beam evaporation. (f) The cross-sectional schematic view of the fabricated top-gated EG FET on 6H-SiC.

FET devices using EG films grown on Si-face semi-insulating 6H-SiC surface were fabricated as shown Fig.S1. The source and drain electrodes were formed by depositing Ti/Au(10nm/50nm) films and performing lift-off process. In order to define the EG channel (10 μm length and 4 μm width) and isolate each FET, the EG film was etched with 100 W O_2_ plasma by using photoresist etch mask. After etching, the photoresist layer was removed with acetone. However, it was confirmed that the photoresist residue (~25nm thick) still remained on the EG channel with the surface profiler (KLA_Tencor) and optical microscope as shown in the inset of Fig.S1(b). It is believed that the photoresist layer gets somewhat hardened during O_2_ plasma etching process, leading to the formation of the photoresist residue. This remaining photoresist residue was used as a seed layer for HfO_2_ film deposition. A 50nm-thick HfO_2_ gate dielectric film was deposited by atomic layer deposition (ALD). Finally, the gate contact electrode consisting of Ti/Au(10nm/50nm) metal stack was formed by evaporation and lift-off processes.

Electrical properties of the fabricated FETs were characterized at room temperature in atmosphere. The field effect mobility is defined as $\mu_{FE}=\frac{L_{ch}}{W_{ch}}\cdot\frac{g_{m}}{C_{G}V_{D}}$ where $L_{ch}$ is the channel length, $W_{ch}$ the channel width, $g_{m}$ transconductance ($dI_{D}/dV_{G}$), and $C_{G}$ channel-to-gate capacitance. In our sample, $C_{G}$ is calculated with the relation of$\frac{1}{C_{G}}=\frac{1}{C_{photoresist}}+\frac{1}{C_{HfO_{2}}}$. $C_{photoresist}$ and $C_{HfO_{2}}$ are determined by $\varepsilon_{0}\varepsilon_{r}\frac{A}{d}$ where $\varepsilon_{0}$ is the vacuum permittivity, $\varepsilon_{r}$ dielectric constant (4.0 for photoresist and 12.0 for HfO_2_), $A$ the channel area, and $d$ the thickness of either photoresist or HfO_2_ layer. The drain-current vs. gate-voltage ($I_{D}-V_{G}$) curves were measured for several different drain voltages ($V_{D}=0.1 V, 0.5 V, 1.0 V$) as shown in Fig.5. At $V_{D}=0.1 V$, the measured peak transconductance is 1.54 mS/mm which is translated to mobility of ~1806 cm^2^/Vs. The on/off ratio is ~4.64.
